# Strategic priorities for TB control in Bangladesh, Indonesia, and the Philippines – comparative analysis of national TB prevalence surveys

**DOI:** 10.1186/s12889-020-08675-9

**Published:** 2020-04-25

**Authors:** Neeraj Kak, Krishnapada Chakraborty, Swati Sadaphal, Hala Jassim AlMossawi, Marianne Calnan, Begum Vikarunnessa

**Affiliations:** 1 University Research Co., LLC (URC), Chevy Chase, MD USA; 2 University Research Co., LLC (URC), Manila, the Philippines; 3 University Research Co., LLC (URC), Dhaka, Bangladesh

**Keywords:** Tuberculosis, Prevalence, Incidence, End TB

## Abstract

**Background:**

Philippines, Indonesia, and Bangladesh are three high tuberculosis (TB) burden countries in Asia which account for 18% of the estimated global TB incidence (1.8 million) and 15% of TB related deaths (192,000). In 2017 alone, approximately 785,000 of the incident TB cases in these countries remained missing, including diagnosed but not notified.

**Methods:**

We reviewed the published data from the most recent TB prevalence surveys conducted in Bangladesh, Indonesia, and the Philippines. The prevalence rates established by the surveys were used to estimate the disease burden of these countries for 2017. The Global TB Report 2017 and World Health Organization’s (WHO) global TB database were sourced for collection of incidence and notification data by age groups and types of TB to estimate prevalence to notification gaps 2017.

**Results:**

According to the surveys, the estimated prevalence rates of bacteriologically confirmed TB and smear-positive TB are 287 and 113 for Bangladesh (2015–16), 759 and 256 for Indonesia (2013–14) and 1159 and 434 for the Philippines (2016) per 100,000 population over the age of 15 years. The overall national TB prevalence estimates for all forms is 260 for Bangladesh, 660 for Indonesia, and 970 for the Philippines (2016). Compared with the incidence rate, the proportion of total notified cases is 67% for Bangladesh, 52% for Indonesia, and 55% for the Philippines. Bangladesh has been able to detect almost 100% of the prevalent pulmonary TB, while Indonesia and Philippines have detected only 30 and 22% of these infectious cases respectively. Although notification has been improving over the years, there is no impact on the incidence rate since a large proportion of the undiagnosed cases, and delayed diagnosis continue to feed the transmission process.

**Conclusion:**

The surveys have provided data that is critical for developing realistic strategies for these countries to eliminate TB. In general, this paper recommends interventions for strengthening diagnosis of pulmonary TB, implementing targeted communication programs and active case finding to reduce patient level delays, expanding public-private partnership to increase access to TB services, using rapid diagnostics, and providing social protection for vulnerable populations. These measures can accelerate these countries’ progress towards achieving End TB goals.

## Background

Tuberculosis (TB) is a major global public health threat. In 2017, about 10 million men, women and children developed TB disease, and 1.3 million died of TB [1]. Three countries in Asia: the Philippines, Indonesia, and Bangladesh account for 18% of the estimated TB incidence (1.787 m) and 15% of TB related deaths (192,000) globally. In 2017 alone, approximately 785,000 of the incident TB cases in these countries, approximately 21% of the global incidence cases, remained missing including diagnosed but not reported. The detection and treatment of Rifampicin Resistant TB (RRTB) accounts for only a small fraction of the estimated cases.

Although TB is a preventable and curable disease, the situation in these countries remains virtually unchanged over the years marked with slow progress and no apparent breakthrough in controlling the epidemic in the foreseeable future. These countries have adopted End TB goals, including targets for 95% reduction in TB deaths and 90% reduction in TB incidence by 2035 compared with 2015 levels with interim milestones to achieve for 2020, 2025 and 2030 [2]. Following United Nations High-Level TB Meeting (UNHLM), the Stop TB Partnership has produced TB diagnosis and treatment targets for 2018–2022 by each country [3]. Reaching the cumulative five-year UNHLM targets for ending TB requires a 45% increase in diagnosis and treatment for Bangladesh, 93% increase for Indonesia and 68% increase for the Philippines over the cumulative notifications that these countries reported during the pre-UNHLM five-year period (2014–2017). In order to reach these targets, the countries will need to reaffirm their commitment to national TB control initiatives with data-driven, effective strategies.

The objective of this paper was to review and analyze available information to understand the whole spectrum of TB disease burden in Bangladesh, Indonesia, and the Philippines. The paper also aimed to discuss strategic options that might be considered to bolster the national TB control initiatives towards achieving the End TB targets. These countries share many similarities concerning population dynamics, epidemiological characteristics, disease transmission patterns, TB control strategies, climatic conditions, exposure to social and biological risk factors, care-seeking practices, and resource mobilization for TB control and prevention. These three countries have completed repeat national TB prevalence surveys following World Health Organization (WHO) guidelines [4] in the past 5 years. The survey provides reliable measures for assessing the trend in disease burden in comparison with the previous surveys that these three countries conducted between 2004 and 2009. The availability of the survey results, including epidemiologic and behavioral data, was an essential reason for combining these three countries for this review. The survey results became available when the need for reliable estimates of the TB disease burden, notification gaps, and challenges was critical for these countries to inform strategic planning for transitioning from control phase to elimination of TB as espoused in the End TB strategy and the Sustainable Development Goals (SDGs).

## Methods

This review is based on published data from the most recent TB prevalence surveys conducted in Bangladesh [5], Indonesia [6] and the Philippines [7]. The prevalence rates established by the surveys were used to estimate the disease burden by bacteriologically confirmed TB (BCTB), smear-positive TB and all forms of TB for Bangladesh, Indonesia, and the Philippines for 2017. The Global TB Report 2017 and WHO’s global TB database were sourced for collection of incidence and notification data by age groups and types of TB. Data from WHO were downloaded into an Excel database. Univariate analyses were used to estimate prevalence to notification gaps by BCTB and smear-positive TB for 2017. Analysis of the results of the diagnostic tools used in the prevalence surveys, e.g., smear microscopy, chest X-Ray, GeneXpert mycobacterium TB/rifampicin (MTB/RIF) and culture, were conducted to understand their effectiveness and recommend strategies that the respective countries may consider for maximizing the cost-effective utilization of available diagnostics to increase detection.

A comparative analysis of the direct and indirect factors was carried out to understand the variations in disease burden and trend in these three countries. For this purpose, we pulled survey data on care-seeking for TB services by survey participants and collected data on other risk factors for TB from available sources for all three countries. We reviewed online published research studies and reports to explain the underlying causes for care-seeking behavior, their impact on service utilization, and to support strategic considerations. Also, we collected available data and reviewed literature to investigate the influence of various socioeconomic determinants, including economic, behavioral and biological risk factors that influence the care-seeking and utilization of TB services, and disease transmission.

## Results

### Disease burden

The surveys, conducted following WHO guidelines, provide reliable prevalence estimates of the bacteriologically confirmed TB and smear-positive TB cases by age, sex, and urban and rural classifications. The repeat survey TB prevalence estimates are significantly above the confidence interval of the previous surveys for Indonesia and the Philippines and within the range for Bangladesh. According to the surveys, the estimated prevalence rates of BCTB and smear-positive TB are 287 and 113 for Bangladesh (2015–16), 759 and 256 for Indonesia (2013–14) and 1159 and 434 for the Philippines (2016) per 100,000 population over 15 years of age. The overall national TB prevalence estimates for all forms is 260 for Bangladesh, 660 for Indonesia, and 970 for the Philippines (2016). The overall disease burden calculated for these three countries in 2017 is based on the prevalence rates. This information is presented in Table 1.

**Table 1:** TB Disease Burden of Bangladesh, Indonesia and Philippines, 2017

Country	Prevalence all forms all ages*	Prevalence of Smear + TB 15 + *	Prevalence of BCTB 15 + *	Incidence all forms**	MDR/RR TB among Pulmonary**	Incidence Child TB (0–14)**
Bangladesh	429,800	134,000	340,900	364,000	5800	35,000
Indonesia	1,742,000	506,800	1,502,800	842,000	12,000	49,000
Philippines	1,018,000	305,300	815,300	581,000	20,000	71,000

*Estimate using the prevalence rates of respective countries for the total population. **WHO Global TB Report 2018

In all three country settings, there was a similarity in the pattern of the distribution of TB in the general population regarding age, sex and habitation as well as the effect of some social and biological risk factors for TB. Additionally, the surveys defined the reach of TB services in the general population and the health-seeking behavior of persons when they have TB symptoms or the disease. In all three countries, the typical pattern illustrated TB prevalence rates were significantly higher among older age groups, in males, and urban areas. According to survey estimates, the proportion of BCTB out of the total estimated prevalence among 15+ years people is 79% in Bangladesh, 86% in Indonesia, and 80% in the Philippines. Prevalence of smear-positive TB is approximately 30% of the estimated total prevalence of all forms in these countries.

Compared with the prevalence, notification of BCTB (for all ages) is 58% for Bangladesh, 23% for Indonesia and 42% for the Philippines. The prevalence survey also estimated notification gaps by different age groups. The highest notification gap was observed in the oldest population in Bangladesh and Indonesia while this gap was highest for the younger group (15–24 years) in the Philippines. The prevalence to notification gap is significantly higher for male for all age groups across these countries.

### Notification

**Fig. 1:**
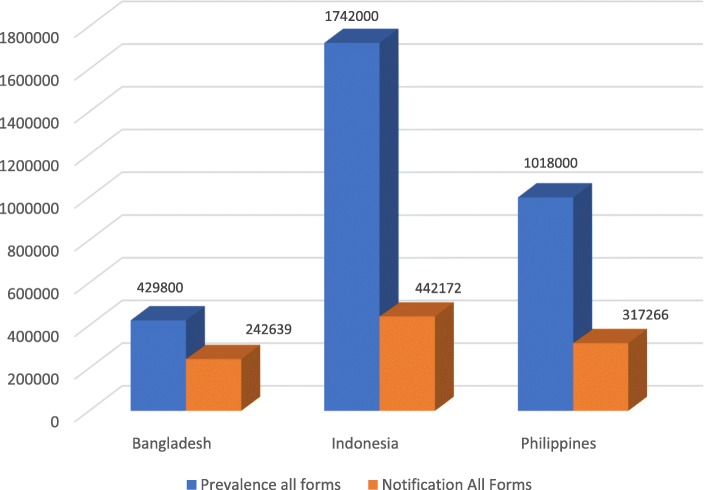
Prevalence to Notification of TB New and Relapse 2017

**Fig. 2:**
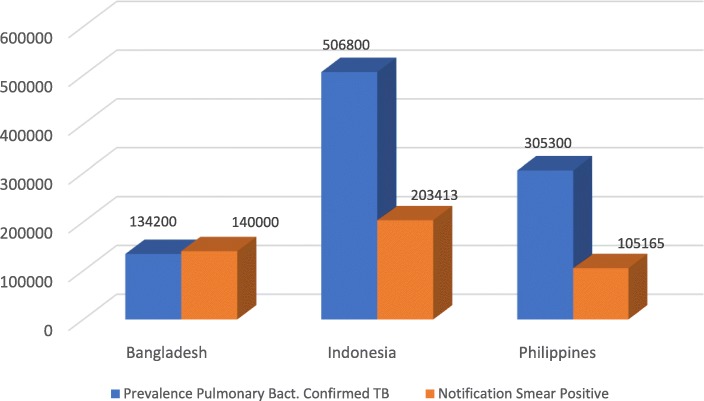
Ratio of Prevalence of Pulmonary Bact. Confirmed TB: Notification of Smear Positive TB

The total notification of new and relapse TB cases compared with 2017 prevalence estimates shows considerable detection and reporting gaps for all three countries (Fig. 1). The proportion of total notified cases out of overall prevalence for all age groups is 56% for Bangladesh, 25% for Indonesia, and 31% for the Philippines. Compared with the incidence rate, which is commonly used for program performance measurement, the proportion of notified cases is 67% for Bangladesh, 52% for Indonesia, and 55% for the Philippines. Figure 2 presents the ratio of estimated prevalence of bacteriologically confirmed TB among 15+ population to notification of pulmonary bacteriologically confirmed TB (smear-positive TB) for 2017 including children 0–14 years. The WHO TB country database, which is our source of data, does not separately report pulmonary TB notification for children. The data indicates that a large proportion of infectious smear positive cases remain undetected in Indonesia and the Philippines compared to Bangladesh. Notification of RRTB is very low in all three countries with laboratory confirmation of only 16% for Bangladesh, 42% for Indonesia and 32% for the Philippines, and even lower rates for treatment initiation.

**Fig. 3:**
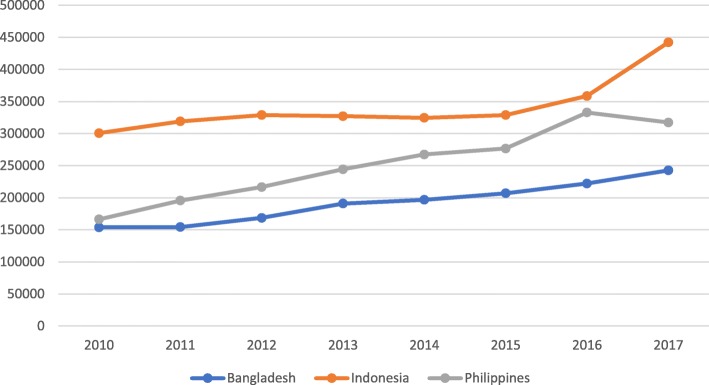
Trends in Notification of All Cases 2010–2017

Analysis of notification data for the last 8 years up to 2017, as presented in Fig. 3, shows an increasing trend over the period. The average annual percentage of increase in notification is 8.2% for Bangladesh, 6.7% for Indonesia and 13% for the Philippines. However, this increase in notification is making no impact on the incidence rate since a large proportion of the undiagnosed cases and delayed diagnosis of the notified cases continue to feed the transmission process.

### Care-seeking for TB services

We collected and analyzed the survey data to understand the care-seeking pattern of people with symptoms suggestive of TB (Table 2). In all three countries, the overall care-seeking pattern of a large proportion of people with symptoms suggestive of TB is characterized by either not seeking care or relying on self-treatment, drug stores and untrained informal providers for initial treatment. Utilization of public health system for TB is a secondary choice for most of the presumptive TB patients in all three countries.

**Table 2:** Care seeking for TB services by prevalence survey participants with TB symptoms

Care seeking	Bangladesh [6]	Indonesia [7]	Philippines [8]
Public hospital/health facility	7%	13.8%	12%
Private provider/clinic	8%	7.9%	7%
Pharmacy/drug store/village doctor	34%	30.8%	0%
Self-treatment	2.4%		40%
Did not seek care	48.2%	43.1%	41%

### Other risk factors

The surveys have identified several risk factors, such as previous TB treatment, older age group, being male, diabetes mellitus, smoking, indicators of poverty, and urban dwellers that are significantly associated with having pulmonary TB. Among the survey participants in Bangladesh, 65% of the BCTB cases were found among current and previous smokers compared to 35% among nonsmokers. This rate is 66 and 34% for Indonesia and 67 and 33% in the Philippines between these two groups. The survey findings also show that the proportion of pulmonary TB cases was significantly higher among those with diabetes, previous history of TB and living with TB patients, compared to those who did not report these risk factors.

## Discussion

The subject countries of this study have a lot in common in terms socioeconomic, demographic, epidemiologic and climatic conditions. Yet, the survey findings of the TB prevalence rates being much higher in Indonesia and the Philippines than in Bangladesh raises questions of the underlying factors that are direct or indirect determinants of the disease burden. According to the survey findings, prevalence of smear-positive TB (pulmonary bacteriologically confirmed TB) is 2.5 times higher in Indonesia and 3.3 times higher in the Philippines compared to Bangladesh. On the other hand, notification of smear-positive TB is only 30 and 22% in Indonesia and the Philippines while Bangladesh has reported notification of almost all the infectious cases. This huge under-detection of the infectious form of TB is perhaps a significant driver of TB transmission in Indonesia and the Philippines. According to the survey, the prevalence of smear-positive TB among Filipinos aged 15–24 is 7.3 times more than their Bangladeshi counterparts. This high prevalence among early age group is indicative of new infections spreading the disease. The notification of child TB as a proportion of total notification is 4% for Bangladesh, and 12% for Indonesia and the Philippines. The child TB notification can be considered as a sentinel of the higher level of ongoing transmission happening in these two countries.

Delayed diagnosis and treatment of TB is associated with significantly increased risk of pulmonary sputum smear positivity and pulmonary cavitary disease [8]. The prevalence surveys in all three countries found a large proportion of the participants with one or more TB symptoms did not seek any care. Of this group, 31% in Indonesia and 41% in the Philippines applied self-medication. In Bangladesh, 34% of those having TB symptoms sought care from drug sellers and village doctors many of whom do not have the basic knowledge about TB treatment. The majority of BCTB cases identified during the survey in these countries, although being TB symptomatic, were not diagnosed before and not under treatment during the survey. This clearly indicates significant delays in care-seeking, diagnosis and treatment. A study conducted in Bangladesh, awaiting publication, found a total mean delay of 77 days, including 57 days of patient delay, 18 days of diagnostic delay, and 2 days of treatment delay [9]. Another study conducted in China found that 95% of patients self-presented for evaluation of illness after a median 58 days of delay after symptoms began [8]. Addressing patient-level delays is challenging but crucial for transmission control.

Several factors, including poor knowledge about TB, stigma and discrimination, symptom recognition, barriers to access to services, initial care-seeking from informal providers or self-medication, contribute to delays at patient-level and systems-level, including diagnosis and treatment. Meta-analysis conducted to determine the factors associated with delay in Asian countries highlights that socio-demographic factors, including low level of income and unemployment, were positively associated with long patient delays [10]. While strategies to address delays will vary by country and local context, focus should be given on massive communication programs that target high-risk vulnerable populations living in urban slums and hard-to-reach areas. These priority measures should promote early care-seeking for TB services and strengthen active search, including systematic contact investigation for early case finding, linking presumptive cases to rapid diagnostics and treatment initiation. This must be complemented with efforts to streamline the diagnostic and treatment initiation processes, including monitoring turn-around and other procedural response times at different levels to reduce health system delays.

The countries need to significantly increase access to detection of both pulmonary smear-positive and smear-negative as well as extra-pulmonary TB. An assessment of patient pathways conducted in Indonesia revealed that only 20% of patients encountered diagnostic capacity at the location where they first sought care [11]. There is no cut and dried answer to how these countries can achieve this goal for the detection of the enormous number of missed TB cases. The survey findings show that AFB microscopy, as already known, was not sensitive for detection of BCTB. The GeneXpert MTB/RIF and chest X-Ray proved very useful and contributed to the detection of the majority of pulmonary positive and negative TB cases for the prevalence surveys in all three countries. The countries need to realign their strategies with focus on expanding access to radiology and GeneXpert to ensure rapid diagnosis and treatment initiation. Instead of testing all presumptive cases by GeneXpert, countries may consider introducing a triage system for pre-GeneXpert screening and ensuring better utilization of the technology with effective referral linkages to reduce the cost of diagnosis.

Notification of large number of childhood TB, particularly in Indonesia and the Philippines, suggests that a more systematic contact tracing of household contacts, including children and adults of active pulmonary TB patients, is essential. Contact tracing is a novel way to identify presumptive TB cases at an early stage and halt transmission and initiate preventive therapy for eligible members to stop disease progression. Despite many benefits of this approach, progress has been slow in implementing systematic contact tracing nationwide. In the Philippines, strain typing of M. tuberculosis isolates from household contacts indicates that transmission of TB does not necessarily occur directly from the index patient; rather, that community-based transmission also frequently occurs [12]. The evidence suggests that effective contact investigation be expanded to cover workplaces and social meeting points of the index cases. The detection of a significantly higher proportion of TB cases among those who ever lived with TB patients or who ever diagnosed with TB, lends credence to expansion of active case finding or contact tracing targeting these groups.

Universal access to TB services, which is critical for rapid finding of the missing TB cases, cannot be achieved without expanding the network of private facilities and providers that are located closest to those who need the service. Initial care-seeking from the private sector is 84% in Bangladesh, 74% in Indonesia and 70% in the Philippines [13]. The prevalence survey also found that use of private sector for TB services is a first choice of a large proportion of TB symptomatic persons. Unless these providers strengthen their capacity to provide quality TB services following national guidelines, reducing diagnosis and treatment delay and incidence of drug resistance as a result of incomplete and incorrect treatment will remain elusive. Strategies to engage with the private sector will need differentiated approaches to local contexts in consideration of care-seeking patterns, capacity of the public sector to coordinate and provide oversight, and capacity of private providers. Countries may also consider strategic purchasing of TB services from private providers while linking the services to quality standards and notification requirements.

Many new cases of TB are attributable to poverty, undernourishment, HIV infection, smoking, diabetes, and alcohol use, which are indicators in the SDG framework for TB. High Out-of-Pocket expenditures for health care and absence of social protection schemes in all resource-poor countries are significant barriers to universal coverage of TB services. A recent modeling study shows that ending extreme poverty will result in 33% reduction in global incidence of TB and expanding social protection coverage will result in 76% reduction in incidence by 2035 [14]. While the result is very inspiring, political will and domestic resource mobilization for implementation of strategies will need to be ramped up. Given dynamic leadership and commitment, multi-sectoral collaboration will be an effective approach for integrating TB with other non-communicable diseases and socioeconomic programs of other ministries and expansion of social protection for vulnerable population.

The findings from the prevalence surveys have enormous implications for redefining TB control strategies and priorities. The survey data can be used to accelerate changes in policies, algorithms, implementation approaches and resource mobilization. Many opportunities for bringing these changes were missed in the past for lacking in leadership and timely initiative. An independent assessment conducted for 16 countries that had completed prevalence surveys found little progress in bringing desired policy changes [15]. The data is now available for the countries to build momentum around TB control priorities through targeted advocacy and communication of the survey results with different stakeholders, implementers and beneficiaries.

## Conclusion

The completion of the repeat national prevalence surveys in Bangladesh, Indonesia and the Philippines, has given a clear picture of the overall TB disease burden, trends of program performance, those most affected by this disease, effectiveness of diagnostics, and care-seeking pattern of survey participants, including those diagnosed with TB and those screened negative for the disease. The prevalence surveys are intended for the development of realistic strategies for the countries to eliminate TB. Our effort through this paper is to expand the base of evidence with additional data and analyses of the underlying factors to support the countries in their strategy development initiatives. In general, this paper recommends interventions for strengthening diagnosis of pulmonary TB, implementing targeted communication programs and active case finding to reduce patient-level delays, expanding public-private mix to increase access to TB services, using rapid diagnostics, and providing social protection for vulnerable populations. These measures can accelerate the countries’ progress towards achieving End TB goals.

The comparative analysis also shows a need for similar data at sub-national level to see what is working and what needs to be changed to achieve national and global targets for ending TB. However, this would require creative approaches to data collection – using sentinel sites – to produce generalizable results. In addition, we believe there is a need to do similar analysis using the recently completed TB prevalence surveys in Africa.

## Data Availability

Analysis is based on published reports available on the web. The survey reports are available on the web or from the authors. Access to WHO Global TB data base is also available on the web. No prior approvals are needed to use the data.

## Abbreviations

BCTB: Bacteriologically confirmed Tuberculosis
MTB/RIF: *Mycobacterium Tuberculosis*/Rifampicin
RR: Rifampicin Resistant
RR TB: Rifampicin Resistant Tuberculosis
TB: Tuberculosis
SDG: Sustainable Development Goals
UNHLM: United Nations High Level Meeting (held in New York in 2018)
WHO: World Health Organization
